# The level and trend of road traffic injuries attributable mortality rate in Iran, 1990–2015: a story of successful regulations and a roadmap to design future policies

**DOI:** 10.1186/s12889-021-11721-9

**Published:** 2021-09-22

**Authors:** Mehran Shams, Farnam Mohebi, Kimiya Gohari, Masoud Masinaei, Bahram Mohajer, Nazila Rezaei, Ali Sheidaei, Sara Khademioureh, Moein Yoosefi, Milad Hasan, Bahman Damerchilu, Ayyoob Jafari, Farshad Farzadfar

**Affiliations:** 1 Non-Communicable Diseases Research Center, Endocrinology and Metabolism Population Sciences Institute, Tehran University of Medical Sciences, Tehran, Iran; 2 Haas School of Business, University of California, Berkeley, California USA; 3 Department of Biostatistics, Faculty of Medical Sciences, Tarbiat Modares University, Tehran, Iran; 4 Department of Epidemiology and Biostatistics, Tehran University of Medical Sciences, Tehran, Iran; 5 Endocrinology and Metabolism Research Center, Endocrinology and Metabolism Clinical Sciences Institute, Tehran University of Medical Sciences, Tehran, Iran; 6 Faculty of Electrical, Biomedical and Mechatronics Engineering, Qazvin Branch, Islamic Azad University, Qazvin, Iran

**Keywords:** Road traffic injuries, Iran, Trend, Mortality, Sustainable development goal

## Abstract

**Background:**

Road-Traffic-Injuries (RTIs) are predicted to rise up to the fifth leading cause of worldwide death by 2030 and Iran has the third highest RTIs mortality among higher-middle income countries. Although the high mortality of RTI in Iran is a warning, it provides the opportunity to indirectly assess the implemented RTI-related regulations’ effectiveness via high-resolution relevant statistics and, hence, Iran could serve as a guide for countries with similar context. In order to do so, we utilized this study to report the time and spatial trends of RTIs-related mortality in different age and sex groups and road user classes in Iran.

**Methods:**

Based on the national death-registration-system (DRS), cemeteries data, and the demographic characteristics, and after addressing incompleteness, we estimated mortality rates using spatiotemporal and Gaussian process regression models. We assessed Pearson seatbelt and helmet use and RTIs-attributable Age-Standardized-Morality-Rate (ASMR) associations. We also predicted RTIs-death-numbers, 2012–2020, by fitting a Generalized Additive Model to assess the status of achieving relevant sustainable development goal (SDG), namely reducing the number of RTIs-related deaths by half.

**Results:**

Overall RTIs-attributable death and ASMR at the national level increased from 12.64 [95% UI, 9.52–16.86] to 29.1 [22.76–37.14] per 100,000 people in the time period of 1990–2015. The trend consisted of an increasing segment in 1990–2003 followed by a decreasing part till 2015. The highest percentage of death belonged to the three-or-more-wheels motorized vehicles. Pedestrian injuries percentage increased significantly and the highest mortality rate occurred in 85 years and older individuals. Low prevalence of seatbelt and helmet use were observed in provinces with higher than the median ASMR due to the relevant cause of each. RTIs-attributable death number is expected to reduce by 15.99% till 2020 which is lower than the established SDG goal.

**Conclusions:**

Despite the observed substantial moderation in the RTI-ASMR, Iran is till among the leading countries in terms of the highest mortality rates in the world. The enforced regulations including speed limitations (particularly for elder pedestrians) and mandatory use of seatbelt and helmet (for young adult and male drivers) had a considerable effect on ASMR, nevertheless, the RTI burden reduction needs to be sustained and enhanced.

**Supplementary Information:**

The online version contains supplementary material available at 10.1186/s12889-021-11721-9.

## Background

In 2018, Road Traffic Injuries (RTIs) were the leading cause of death for children and young adults in the world; RTIs caused many countries to lose approximately 3% of their gross domestic product and it affected men, the main workforce of countries, 3 times more than women [1]. It is predicted that by 2030, RTIs will upgrade to the fifth leading cause of death worldwide mainly owned to the observed, and predicted to be continued, increasing burden in developing countries [2, 3].

Iran is the third higher-middle income country in the global rankings in terms of RTIs mortality rates [1]. The Iranian forensic medicine organization, the official source of death announcement, reported 16,872 and 16,584 people died in 2014 and 2015 due to RTIs in Iran, respectively, with an estimated cost of 6.64% of the gross national income in 2013 [4, 5]. According to the Global Burden of Disease report in year 2019 (Supp Figure Adapted from GBD), the number of deaths due to RTIs in Iran did not significantly decrease and it is comparable to the deaths caused by total cancers and also all communicable diseases in Iran [6]. It is important to mention that Iran has considerable number of highways and mountain inter-city roads in the north and west part of the country. Besides, fair number of suburb roads lack lights, active police and active emergency stations [7]. Despite the importance of RTIs and the demand for accurate statistics to inform further policy makings, reports have been mainly national, do not reflect the subnational variations and implemented strategies’ effectiveness, encounter incompleteness, and are derived from data with questionable quality [8]. Additionally, the few available reports are not consistent in their statistics. For example, two studies in 2020 studies reported that the RTIs reports and databases in Iran are problematic due to set of underreported and duplicated information [9, 10]. Alongside the absence of accurate point estimations, the time trend reports consist of small number of data points, lack classification accuracy and completeness in their data source, and most importantly they cover short periods of time [11–13].

Therefore, we reported the high-resolution time and spatial trends of RTIs-related mortality to provide information and to better understand the context, regulations, and policies that might have caused the observed patterns. Besides, we believe the detailed estimations could help national and global stakeholders to understand Iran, as a developing country, for further policy planning. In order to do so, we used the National and Subnational Burden of Diseases, Injuries, and Risk Factors (NASBOD) study, conducted to estimate the burden of diseases from 1990 to 2015 in Iran, to estimate the spatial and temporal trends of mortality rate due to RTIs in Iran by age and sex groups in different classes of road users [14, 15]. We believe the provided details would help to determine national health priorities, plan for effective prevention strategies, assess previously established policies’ success, and provide guidance for the countries with similar setting regarding what policies are more likely to decrease RTI burden.

## Methods

This study builds up on NASBOD study and, herein, we briefly review the statistical methods to estimate ASMR attributed to RTIs [14–17]. Although it is not the specific aim of this study, it is worth mentioning that NASBOD study was aimed to utilize all available published and unpublished data sources for estimating the burden of 291 diseases and 67 risk factors from 1990 to 2015 at national and subnational scale in Iran. The aforementioned notion emphasizes why NASBOD could be the key source for supporting this study. All Iran maps are downloaded from https://www.openstreetmap.org/ and further loaded in the R software to draw the figures.

### Data source

The primary data was gathered from Iranian Ministry of Health and Medical Education death registration system (DRS) and Tehran and Isfahan cemeteries (two provinces located in the center of Iran that were not supported by DRS). These datasets are not uploaded on any public data source to be cited and they were directly received from the previously mentioned organizations with the permission to use for this research. As this study used previously collected data, we were limited by the duration the data covered and we were not able to gather additional data. Thus, in order for us to extract the time trends despite the limitation, we were required to use prediction models, described in details in the next section. The age and sex distribution of the Iranian population was extracted from the national census reported by statistical center of Iran [18]. Years of schooling and wealth index were extracted from the Household Expenditure and Income Surveys (HHEI) for the equivalent years [18]. Census and IHME datasets are publicly available on https://www.amar.org.ir/english. Missing data on sex and age was imputed with multiple imputation approach in Amelia package in R statistical software to address incompleteness and misclassification. If the recorded cause of death was uncertain, the uncertain deaths were redistributed to the probable cause using proportions of age and sex combinations in each year. Cause-specific fractions related to each age, sex, province, and year combinations were calculated using mixed effect and spatio-temporal models [19]. Death distribution methods (DDMs), including Generalized Growth Balance (GGB) and Synthetic Extinct Generation (SEG), were utilized to address the incompleteness of the death registration system for adult mortality estimation. Full (or complete) birth history (FBH) and summary birth history (SBH) were used to take into account incompleteness and measure child mortality rates [17]. The individual-level data is available upon request, submitted to and approved by the corresponding author. The aggregated data is freely accessible on the webpage https://vizit.report/panel/nasbod/en/main.html#/treemap .

### Causes of deaths

The included categories of causes were based on the Global Burden of Diseases study (GBD) cause of death categorization for road injury including pedestrian injury by road vehicle (categorization code: C.1.1.1), pedal cycle vehicle (C.1.1.2.), motorized vehicle with two wheels (C.1.1.3.), motorized vehicle with three-or-more wheels (C.1.1.4.), and other types of road and transport injuries (C.1.1.5. and C.1.2.) [20]. The equivalent categories of ICD-10 coding, transformed to GBD coding by a physician and verified by a senior physician, were road injury (V01-V04, V06, V09, V10-V19, V20-V29, Y85.0, V30-V79, V87.2-V87.3, V80, V82), pedestrian injury by road vehicle (V01-V04, V06,V09), pedal cycle vehicle (V10-V19), motorized vehicle with two wheels (V20-V29), motorized vehicle with three-or-more wheels (V30-V79, V87.2-V87.3), and other types of road and transport injuries (V80, V82, V05,V81, V83-V86, V88.2, V88.3, V91, V93-V98) [21]. The recorded codes were based on the physician’s diagnosis who completed the death certificates.

### Statistical modeling

Mortality rates were age-standardized for all ages with direct method (using national and sub-national population in 2015 of Iran as standard population) and “epitools” package in R statistical software [22]. Age-standardized mortality rate (ASMR) was chosen as the unit of report to help with picturing the trends and performing comparisons because this indicator adjusts for differences in populations [23]. The average annual percentage change (AAPC) was calculated by Joinpoint regression model as a summary measure of the overall observed variation and the existing temporal trend regarding ASMRs. Briefly, we used a two-stage modeling based on spatiotemporal and Gaussian process regression models (GPR) to estimate mortality rates. The age-spatiotemporal model was used to address the misalignment in the age, space, and time of data, then GPR was employed to extrapolate all-cause (age- and sex-specific) mortality rates [24, 25]. The fraction of each cause of death was applied on the all-cause mortality and extrapolated using the spatiotemporal model [19]. To address misclassification, and after the initial computation of all-cause mortality rates, we divided the deaths into cause-specific rates in proportion to the cause fractions extracted from the original data [15]. The provincial range of ASMR was calculated in each year by subtracting the lowest provincial ASMR from the highest. All graphs and maps were created by R statistical software version 3.1.2 (R Foundation for Statistical Computing, Vienna, Austria).

### Correlates of cause specific ASMR

Age-standardized prevalence of seatbelt and helmet use were gathered from stepwise approach to non-communicable disease risk factor surveillance (STEPS) 2016 survey in Iran [26–28]. The highest tertile of prevalence was considered as good compliance and the lowest as bad compliance. The association of seatbelt using prevalence and RTIs-attributable ASMR caused by motorized vehicle with three-or-more wheels was assessed at provincial level using Pearson correlation. The same analysis was done for helmet use prevalence and the ASMR caused by pedal cycle vehicle and motorized vehicle with two wheels.

### SDG goal achievement assessment

The sustainable development goal 3 depicts its target as “SDG target 3.6: by 2020, halve the number of global deaths and injuries from road traffic accidents” [29, 30]. Besides, the goal of the Global Plan for the Decade of Action for Road Safety 2011–2020 is to stabilize and then reduce the RTI level by 2020 [31]. Accordingly, we predicted the number of deaths attributable to RTIs from 2012 to 2020 by fitting a Generalized Additive Model (GAM) [32] with number of deaths as the response variable and year as the independent variable. The latter analysis has been implemented using mgcv [33] package in R statistical software (version 3.5.2). We used restricted maximum likelihood (REML) method to estimate the parameters of the model.

## Results

### National level and trend of RTIs-attributable deaths

**Fig. 1:**
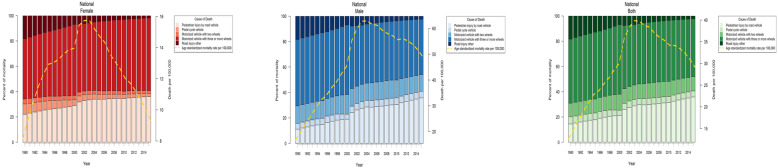
Age-standardized mortality rate and percent of mortality due to road traffic injuries by cause and sex in Iran, 1990–2015

RTIs-attributed ASMR in Iran increased from 1990 to 2003, followed by a decreasing trend. Nevertheless, the ASMR in 2015 was more than 2-fold of the quantity in 1990 as it increased from 12.64 [95% UI: 9.52–16.86] in 1990 to 29.1 [22.76–37.14] per 100,000 people in 2015 (Fig. 1). Despite the similarity of the general time trend of ASMR in males and females, the decrease (from 2003 to 2015) was more prominent in females. The increasing trend of ASMR and the number of deaths due to RTIs were both greater in males than females; ASMR had a 14.4% increase in females (from 8.14 [6.1–10.9] to 9.31 [7.25–11.94] per 100,000) vs the 190% increase in males (from 16.78 [12.67–22.36] to 48.77 [38.19–62.19] per 100,000). Moreover, the number of deaths increased from 1848 to 3722 in females and from 3963 to 19,622 in males (Table 1). As expected, male to female ASMR ratio at the national level changed from 2.06 to 5.23 from 1990 to 2015.

**Table 1:** The number of death and age-standardized mortality rates due to RTI by sex, in Iran, 1990–2015

Sex	1990		1995		2000		2005		2010		2015		Average Annual percent change in ASMR (%)
	Number of deaths	^**a**^ASMR per 100,000 [95% ^**b**^UI]	Number of deaths	ASMR per 100,000 [95% UI]	Number of deaths	ASMR per 100,000 [95% UI]	Number of deaths	ASMR per 100,000 [95% UI]	Number of deaths	ASMR per 100,000 [95% UI]	Number of deaths	ASMR per 100,000 [95% UI]	
Female	1849	8.14 (6.1–10.9)	3081	12.93 (9.9–16.91)	3577	13.95 (10.91–17.88)	4289	14.72 (11.56–18.76)	4052	12.01 (9.42–15.31)	3722	9.31 (7.25–11.94)	0.14 (−0.16–0.43)
Male	3964	16.78 (12.67–22.36)	8018	32.29 (24.91–42.06)	12,369	45.34 (35.78–57.62)	19,709	61.61 (48.56–78.28)	19,809	55.64 (43.86–70.61)	19,622	48.77 (38.19–62.19)	4.11 (3.86–4.36)
Total	5812	12.64 (9.52–16.86)	8018	32.29 (24.91–42.06)	15,946	30.01 (23.63–38.21)	23,998	38.74 (30.51–49.26)	23,861	34.13 (26.88–43.35)	23,344	29.1 (22.76–37.14)	3.12 (2.87–3.37)

^a^*ASMR* Age-standardized mortality rates

^b^*UI* Uncertainty interval

### Causes of RTIs

In 2015, the motorized vehicles with three-or-more wheels were responsible of 57.36 and 43.53% of injuries in females and males, respectively. The second highest fatal RTI occurred in pedestrians for both males (35.80%) and females (36.07%) in 2015 and females in 1990 (23.4%); other types of road injuries were the second highest cause of fatal RTIs among males (16.70%) (Fig. 1). When looking at the general trend of causes of RTI, despite that the motorized vehicles were the highest contributor category, the proportion of pedestrian injuries by road vehicles increased from 1990 to 2015. However, proportion of injuries related to motorized vehicles and pedal cycles were constant. To elaborate more pedestrians, the second highest fatal RTI category in both males and female, we refer to Fig. 1: The proportion of RTI-mortality attributes to pedestrians substantially increased in the years of study both in females and males. In other words, it increased from just being approximately 15% of the total RTI-mortalities in 1990 to almost 23% in 2015. The observed patterns were almost the same in females and males with the two exceptions; motorized vehicles with three-or-more wheels’ proportion increased in females while it slightly decreased in males and the rising trend of pedestrian injuries was larger in males.

### Age trend of RTIs

**Fig. 2:**
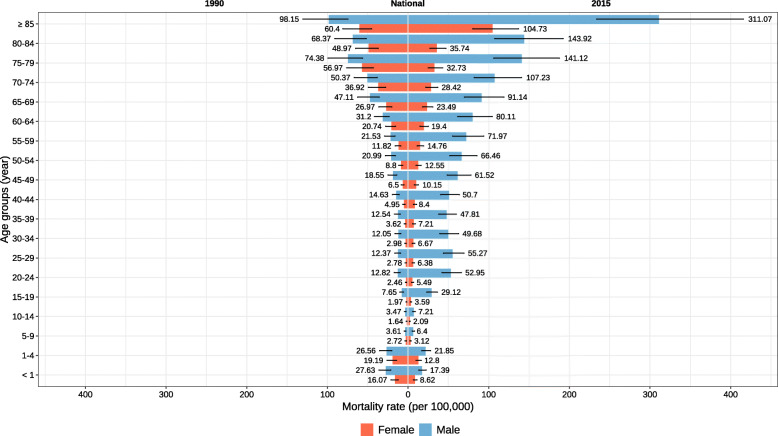
Age-specific mortality rate due to road traffic injuries by sex in Iran, 1990–2015

**Fig. 3:**
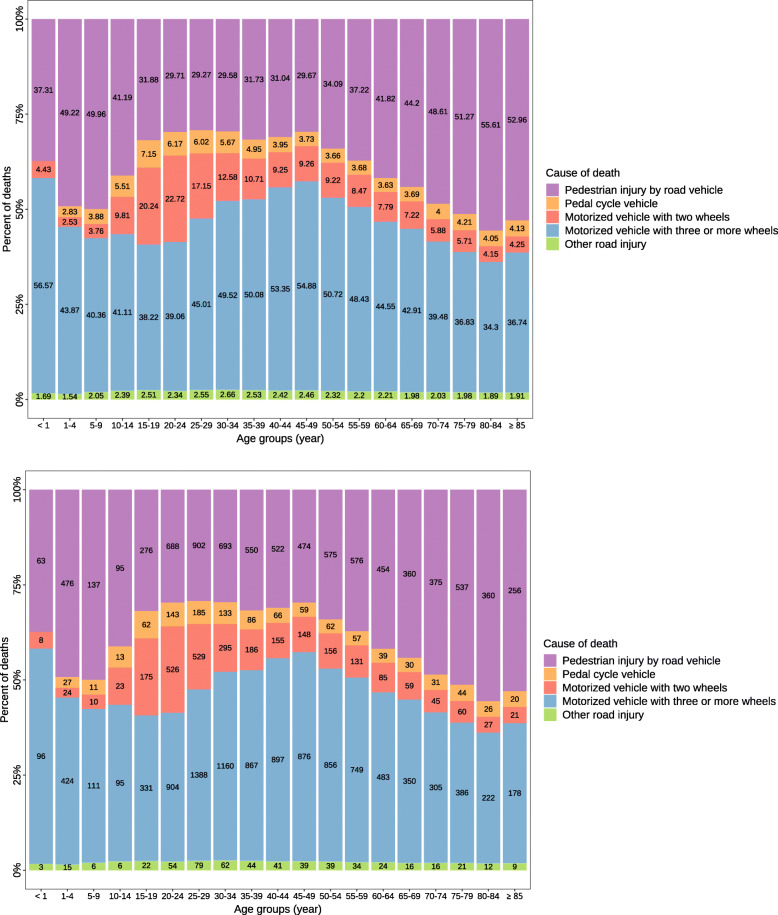
Causes of RTIs-attributable deaths composition by age group in 2015 in Iran

In both genders, the highest mortality rate occurred in 85 years and older individuals in 1990 and 2015 (Fig. 2 and Fig. 3). The mortality rate due to RTIs increased in all age-groups of both genders but under-5 children and the 60–85-year-old females, which presented a decreased ASMR. From 1990 to 2015, the contribution of pedestrian injuries by road vehicle considerably increased in all age groups such that pedestrian injuries became the most prevalence cause of RTI-mortality rate in older ages. When comparing difference in age groups in 2015, inasmuch as the age increased, the contribution of pedestrian injuries decreased until the age of 50. The injuries related to motorized vehicles with three-or-more wheels almost presented a reverse pattern with the highest contribution in less-than-1-year followed by 45–49-year-old age group. There a few other patterns that comes to attention in Fig. 3, a great proportion of RTIs deaths is related to motorized vehicles in ages 15 to 19 (the illegal ages of using these vehicles up to 18 years old); at the ages of 1–4, when children start to walk on their own, we observe a high increase in the proportion of pedestrian injuries; and pedal cycle vehicles, though having almost the lowest contribution to RTI deaths, still has high proportions in almost all age categories. It is worthy of mentioning that almost 50% of the RTI-related mortalities were in pedestrians in age categories 1–4, 5–9, and over 65 years old. Finally, the contribution of the motorized vehicles with two wheels to deaths was highest in 15–24-year-olds.

### Behavioral associates of RTIs

Most of the provinces with higher than the median ASMR caused by three-or-more wheels motorized vehicles had low compliance of seatbelt (Supplementary Figure 2). Only two of all provinces with higher than median ASMR had acceptable compliance of seatbelt use. The pattern was almost similar for helmet use in provinces in terms of their ASMR caused by pedal cycle and two-wheeled-motorized vehicles (correlation coefficient: − 0.28) (Supplementary Figure 3). In the top five provinces ranked by ASMR due to pedal cycle and two-wheeled-motorized vehicles, only one were in the highest tertile of helmet use prevalence. In comparison, among the five provinces with the lowest ASMR caused by pedal cycle and two-wheeled-motorized vehicles, three of them were in the highest tertile of helmet use prevalence (correlation coefficient: − 0.33). Table 2 provides detailed statistics of the Behavioral associates of RTIs in Iran.

**Table 2:** The correlation of age-standardized prevalence of RTI-related behavior with RTIs-attributable ASMR caused by relevant injury type, at provincial level

Covariates	Female		Male		Both	
	Correlation coefficient	***P***-value	Correlation coefficient	***P***-value	Correlation coefficient	***P***-value
Age-standardized prevalence of Seatbelt usage and RTIs-attributable ASMR caused by motorized vehicle with three or more wheels	−0.41(−0.67, −0.06)	0.02	−0.12(− 0.46, 0.25)	0.51	−0.29(− 0.59, 0.08)	0.12
Age-standardized prevalence of helmet usage and RTIs-attributable ASMR caused by pedal cycle vehicle and motorized vehicle with two wheels	−0.13(− 0.47, 0.24)	0.48	−0.23(− 0.55, 0.14)	0.21	−0.33(− 0.62, 0.03)	0.07

### Subnational statistics of RTIs-attributable deaths

Male to female ASMR ratio at the provincial level was nearly fivefold in most provinces in 2015 and the ratio increased by twice from 1990 in almost all provinces (Supplementary Figure 1). In 2015, the provinces with highest and lowest ASMR were Fars and Qom, respectively. All provinces represented increased ASMR from 1990 to 2015 except Zanjan (1990: 22.24 (15.97–31.04), 2015: 20–97 (15.97–27.47)). The general pattern of Zanjan province was also different as it presented a decreasing trend from 1995, followed by an increase and again another decrease from 2003. In general, the provincial range of ASMR increased from 17.71 in 1990 to 36.63 in 2015. The provincial range of ASMR due to RTIs did not differ substantially in females from 1990 to 2015 (in 1990 the lowest ASMR was 2.67 per 100,000 [1.95–3.67] and the highest ASMR was 15.02 [10.95–20.57]; in 2015 the lowest ASMR was 5.32 [3.96–7.11] and the highest ASMR was 17.05 [13.49–21.49]). However, the provincial differences increased by a 2.56-fold in males (in 1990 the lowest ASMR was 6.21 [4.62–8.38] and the highest ASMR was 30.57 [24.28–38.49]; in 2015 the lowest ASMR was 19.35 [14.54–25.79] and the highest ASMR was 81.84 [65.23–102.4]). Considerably, the ASMR due to RTIs in 2015 decreased from 1990 in some provinces for females, however, all provinces had higher ASMR in males in 2015 than 1990 (Supplementary Table 1). The highest reduction of ASMR was for females in Zanjan and the highest increase was for males in Fars.

### SDG goal achievement

**Fig. 4:**
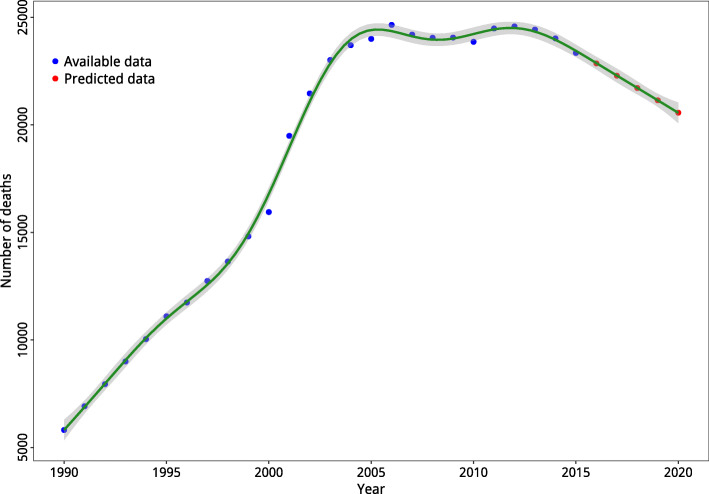
The predicted RTIs-attributable number of deaths till 2020 at national level; evaluating SDG goal achievement

The predicted values for number of deaths are presented in Fig. 4. Compared to the data from 2011, the number of deaths attributable to RTIs is expected to reduce by 15.99% till 2020 which is lower than what is determined by SDG goal (50%).

## Discussion

From 1990 to 2015, the number of deaths and RTIs-attributable ASMR increased at the national level in Iran, overall and in both genders. The ASMR trend consisted of two segments; an increasing pattern from 1990 to 2003 followed by a decreasing trend till 2015 and a relatively larger decrease in females than males. However, the reduction is far less than the expected reduction of SDG goals; the number of deaths attributable to RTIs is expected to reduce by 15.99% from 2011 to 2020, which is lower than what is determined by SDG goal -a reduction to half [29]. The ratio of ASMR in male and in females at the national level changed from 2.06 to 5.23 in this period. The highest percentage of death due to RTIs belonged to the motorized vehicles with three-or-more wheels in all years, however, the proportion of pedestrian injuries by road vehicles substantially increased. In both genders, the highest mortality rate occurred in 85-year-olds and older individuals. While pedestrian injuries by vehicles was the most prevalent cause of RTIs-attributable death in elders, the three-or-more-wheeled motorized vehicles had the most significant contribution in adults and the proportion of two-wheeled motorized vehicles was highest in young adults. At subnational level, the range of ASMR due to RTIs in 2015 increased among provinces from 1990. Finally, most of the provinces with higher than the median ASMR caused by three-or-more wheels motorized vehicles had low compliance of seatbelt use, similar to helmet use in provinces for pedal cycle and two-wheeled-motorized vehicles injuries attributable ASMR.

The estimated RTIs attributable death per 100,000 in Iran was more than 1.5-fold of global estimates and higher than the mean reports of countries with similar Gross Domestic Product (GDP) (upper middle-income countries), region (eastern Mediterranean region and North Africa and middle east), and sociodemographic status (high middle SDI countries) in the same year [34]. However, the gap between the mean estimates of similar countries and statistics of Iran had decreased to the lowest ever [34]. Other previous national, but smaller-scales studies of Iran, reported the same trends with minor variations [11, 35]. It is worthy of mentioning that, some previous studies have recognized the problematized set of underreported and duplicated information in Iranian databases regarding RTIs [9, 10] and we consider the methodology of this study among the best attempts to addressing this issue. The observed pattern in our results follows the same pattern of general reports of national traffic police [36]. Nevertheless, our results are higher than the reports from national forensic medicine, which is, according to previous studies, probably because the deaths that occurs in villages do not have to be reported to the national forensic medicine and they are not completely registered [11]. Additionally, DRS, source of data for the current study, integrates all mortality databases, including forensic medicine and hospitals and validates mortality records by crosschecking the results. As a result, DRS is the most comprehensive mortality database in Iran. In comparison with previous international reports, our results were similar to the GBD reports with respect to the general trends [34], however, the number of attributable deaths was estimated higher in GBD statistics but the difference had decreased over time. When comparing ASMRs, GBD results showed a general decreasing trend both in males and females, which was in agreement with our data since 2003. The probable reason for the wider scope of GBD might be their lack of access to datasets of RTIs-related death numbers in Iran from 1990 to 2000. Secondly, GBD studies included deaths due to consequences of RTIs as well, which resulted in overestimation of the RTIs-attributable mortalities [37, 38]. Therefore, it seems that our report is a more accurate presentation of the current status of Iran regarding RTIs.

Despite the differences in early years of the study in different reports of Iran, patterns were consistent that there was a notable decrease of ASMR due to RTIs since 2003. Generally, RTIs frequency and severity depends on factors in three categories of pre-crash, crash, and post-crash [39]. Although each phase requires relevant prevention and control strategies, it seems that a substantial part of the decrease in ASMR due to RTIs in Iran is attributable to pre-crash prevention strategies. This is the most likely reason particularly considering that the frequency of road traffic crashes have decreased considerably in this period [36]. To elaborate, Iran stands as the 16th largest automobile industry in the world [40]; Iran produced 1,418,550 in 2017. Nevertheless, there is no publicly available report that proves the compliance of domestically designed and produced passenger vehicles with global safety regulations, including frontal impact, electronic stability control, and pedestrian protection [41]. On top of all, the most affordable car has the lowest safety scores in domestic evaluations [42] and is accompanied with higher incidents of road traffic injuries at the accident scene [43]. Besides, the induced and continued economic instability of the country due to sanctions are believed to increase the burden of road accidents in Iran [43]. Meanwhile, there are multiple factors that contribute to the current status in Iran, both increasing the burden of RTIs or impeding its decrease [44]; 1) drivers are likely to perform dangerous behaviors when they are not monitored.; 2) as previously mentioned, cars commonly used by drivers are low in quality and safety.; 3) there is relatively insufficient provision of public transport and, as a result, the tendency to use private cars.; 4) Iran lacks mandatory use of safety equipment including air bags, anti-block braking systems (ABS), and child car seats.; 5) Iran’s roads are not compliant with safety regulations and are low in safety due to inadequate lighting, lack of roadside protection, and sufficient traffic signs for drivers and lack safe routes for pedestrians and cyclists.

Despite all the shortcomings, there has been established regulations that have decreased the RTIs as follows. Therefore, we consider these implemented and established policies and regulation as successful actions because despite the great rising trend of the RTI prior to their implementation, the aforementioned policies and regulations not only stopped the rise but also resulted in a decreasing trend.: In general, there has been 13 policies directly aiming to reduce RTIs in the duration of this study. Nonetheless, “safety belt” policy is considered to be the most effective interventions [45]: this policy was approved by parliament (Majlis) in 1997 and was reviewed and notified for implementation in 2003–2004. The implementation was enforced by eight national organizations directly acting in issues related to RTIs in Iran. Besides, additional regulations were implemented including national speed limit law (maximum 60 km/h in urban areas and 95 km/h in rural areas) [46], and the significant development in the roads network construction in recent years [47]. This notion is supported by the observed parallel higher decrease in number of crashes in inter-city roads than intra-city roads.

Despite the significant decrease of deaths caused by three-or-more-wheeled motorized vehicles, they highly contribute to RTIs attributable ASMR, probably due to the fact that most cars and motorcycles in Iran are not equipped with safety devices that keep the drivers, passengers, and pedestrians safe in different crash situations [1]. Among different contributing factors contributing to pedestrians being vulnerable to RTIs [48], it seems that the increased exposure of pedestrians to cars and motorcycles is the main reason of increased mortality percentage. In other words, the number of cars and motorcycles have increased considerably in the studying years in Iran without equipping them with devices that would reduce the fatality of crashes for pedestrians [43]. On the other, and to our knowledge, there is no specific policy targeting pedestrians, such as safer walking spaces, to immune pedestrians from the increased risk of getting hit by a car [45]. Besides, the burden of RTI for pedestrian significantly differs in terms of number of deaths for females and males; male pedestrians, though being lower in the contribution to the overall RTI-related mortality, are quite higher in number of deaths. This is of great importance for further policy makings and the fact that the higher number could be caused not only but men being more exposed to traffic injuries in Iran due to the culture that men leave the house quite more often than female, but it could also be caused by higher prevalence of risky behavior in males [49].

As previously mentioned, there have been a rapid growth in the number of motorized vehicles in Iran; it increased more than 4.5 fold from 2001 to 2014 and the increase is estimated to continue [50]. Therefore, while the regulations decreased the number of deaths, the increased number of unsafe vehicles had an increasing effect on the deaths and it kept the general ASMR high; presented in results as the adults who died from three-or-more-wheeled motorized vehicle injuries were the most prevalent category among all RTIs-attributed deaths in 2015 in Iran. And finally, the higher decrease in the females could be the result of the inter-gender differences in RTI related behaviors including higher adherence to law in females and the their relatively lower exposure to long unsafe intercity distances [51]. More supporting evidence for the effectiveness of aforementioned policies in reducing the RTI attributable ASMR is that the highest ASMRs are observed in provinces where the inter-city roads have highest lengths and the main proportions of the roads are rural [52].

This study provided the required robust, accurate, and valid evidences based on the updated data sources available in Iran for policy makers. We utilized the most comprehensive dataset of death in Iran which enabled us to analyze the time trend of RTI burden. We also provided ASMR due to RTIs at the sub-national level for specific policy formations and evaluations, namely equity considerations. The patterns among females and males were compared that could help the policy makers to identify populations at higher risk of injuries. And finally, the correlation of safety behaviors was reported with ASMR for the first time in the national scale. However, we faced limitations: Firstly, the limitation of NASBOD study general protocol also applies to this study [14, 15]. In brief, the modeling was conducted only by causes and provinces due to computational constraints. Moreover, we ignored some sources of uncertainty like redistributing and cleaning. Additionally, the death registries, especially in previous years, are prone to incompleteness and misclassifications and were improved by appropriate methodologies as we adjusted for incompleteness and misclassification of the data and calculated uncertainty interval. This study did not calculate the Disability-Adjusted Life Year (DALY) of RTIs, which quantifies the burden from mortality and morbidity. We could not disentangle the deaths happened in intra-cities vs. inter-cities roads to better identify the underlying reason of high ASMR. And finally, we had limited behavioral data according to the type of behavior and time. And last but not least, this study could not exclude other possibilities contributing to the observed trend, such as improvements due to economic growth and improved education. Therefore, we provided a picture of the relevant policies and establishments that could have resulted in the observed trend, however, the RTI is a multifactorial concern in terms of being related to many other sectors, so we remain careful in finalized conclusions as we could not quantitatively disentangle and decompose other possibilities’ contribution.

Taken together, 51.5% of all deaths due to RTIs occurred in 15–44 years old people, the most economically productive members of the population, in Iran. Thus, despite the considerable decrease, the RTIs burden is still high and Iran is far from achieving the SDG goal for 2020 [29]. Therefore, while we should take parallel actions to improve road safety and post-crash responses, the current decrease caused by pre-crash strategies should not be taken for granted and the implemented strategies should be enforced [53]. Moreover, we should be considerate of the high vulnerability of elders to road crashes comparing to other groups and the relatively high ASMR in them. Hence, for designing strategies to maintain the current decrease and even further improve the situation, we should take into consideration the two factors that form the pattern of mortality rates due to RTIs: exposures to road traffic injuries and the probability of death in each crash. According to our results, adult males who drive unsafe cars, especially in inter-city roads, should be prioritized to be the target group of policies to decrease their probability of getting exposed in road traffic injuries and decreasing the impact of the injury is a priority in elders by making the cars and roads safe for pedestrians and improving post-crash cares. However, we recommend sex-specific studies to evaluate the policies targeting women as they are expose to different risk factors. For example, women are less probable to make inter-city trips and they are unlikely to ride motorcycle and bicycles in developing countries. Besides all, the safety of cars is a significant determinant of the burden of RTIs that affects all causes and all ages. Last but not least, to make efficient and sustained changes we must take a multi-dimensional approach that utilizes public education and road and transport related prevention programs alongside with enforcing rules to bring about significant improvements [46].

## Conclusions

This study showed that, albeit the observed substantial decrease in the ASMR due to RTIs, Iran still has one of the highest mortality rates in the world and the current reduction needs to be sustained and amplified to achieve future SDG goals. To reduce the RTIs-related mortality we recommend policy-makers to consider enforcing the current strategies, including speed limitations and safety behaviors of drivers, while developing new plans for susceptible populations including elders and adult males and target crash and post-crash causes of high RTI burden. More specifically, we recommend speed limitations policies to protect elder pedestrians and mandatory use of seatbelt and helmet to protect young adult and male drivers. The impact of urbanization and increasing number of motorized vehicles should not be forgotten as more people are going to be exposed to unsafe vehicles and roads and it increases the demand for medical facilities and pre-hospital emergency services. We also recommend further studies to calculate the DALYs attributable to RTIs, assess the equity of the burden of RTIs in different individuals with different socioeconomical status to recognize and plan for the most vulnerable populations, and design population-specific policies to be efficiently and effectively reduce RTIs burden. On top of all, this study could not evaluate the details of the policies’ effectiveness due to data limitations and we call for further studies to analyze and decompose the effects of different contributing factors to the decreased trend for it to be a better guide for countries dealing with the same concerns.

## Supplementary Information

**Additional file 1: Supplementary Figure 1.** Geographical distribution of age-standardized mortality rate due to road traffic injuries in both sexes in Iran, 1990-2015. Iran’s map is downloaded from https://www.openstreetmap.org/ and further used to draw the figure.

**Additional file 2: Supplementary Figure 2.** The correlation of age-standardized prevalence of seatbelt usage and RTIs-attributable ASMR caused by motorized vehicle with three-or-more in Iran in both sexes at provincial level.

**Additional file 3: Supplementary Figure 3.** The correlation of age-standardized prevalence of helmet usage and RTIs-attributable ASMR caused by pedal cycle vehicle and motorized vehicle with two wheels in Iran in both sexes at provincial level.

**Additional file 4: Supplementary Figure.** Adapted from GBD: Comparing number of deaths attributed to non-communicable diseases, communicable diseases, total cancers, and road injuries in Iran from 1990 to 2019 (Data source: http://www.healthdata.org/data-visualization/gbd-compare).

**Additional file 5: Supplementary Table 1.** The number of death and age-standardized mortality rates due to RTI by sex, in provinces of Iran, 1990-2015.

## Data Availability

Full data would be available upon request submitted to the corresponding author. Aggregated data is available on https://vizit.report/panel/nasbod/en/main.html#/treemap. We gained the permission to access to the death registration system and Tehran and Isfahan cemeteries’ dataset from Iranian Ministry of Health and Medical Education-Network Management Center, Network System Information and Statistics Group’s Manager. Other used datasets are publicly available.

## Abbreviations

RTI: Road-Traffic-Injury
DRS: Death-registration-system
ASMR: Age-Standardized Morality Rate
SDG: Sustainable development goal
NASBOD: National and Subnational Burden of Diseases, Injuries, and Risk Factors study
HHEI: Household Expenditure and Income Surveys
DDM: Death distribution method
GGB: Generalized Growth Balance
SEG: Synthetic Extinct Generation
FBH: Full (or complete) birth history
SBH: Summary birth history
GBD: Global Burden of Diseases study
AAPC: Average annual percentage change
GPR: Gaussian process regression models
STEPS: Stepwise approach to non-communicable disease risk factor surveillance
GAM: Generalized Additive Model
GDP: Gross Domestic Product
DALY: Disability-Adjusted Life Year
