# Supplementary figures and images for: The level and trend of road traffic injuries attributable mortality rate in Iran, 1990–2015: a story of successful regulations and a roadmap to design future policies
Source: BMC Public Health. 2021 Sep 22;21:1722. doi: 10.1186/s12889-021-11721-9 (PMC8459502; doi:10.1186/s12889-021-11721-9)

1990

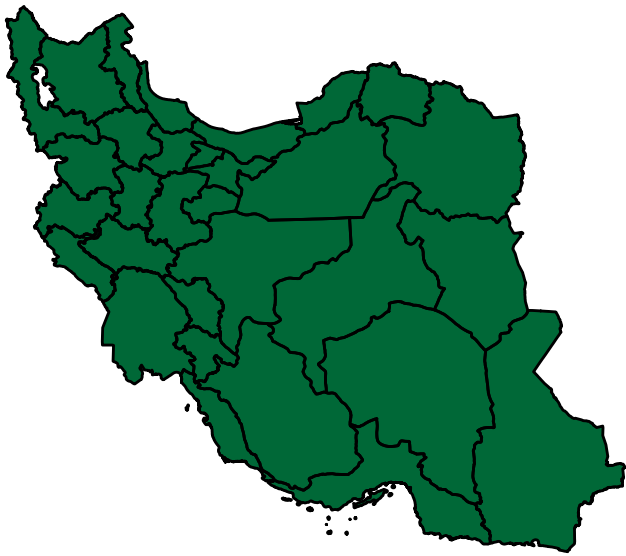

1995

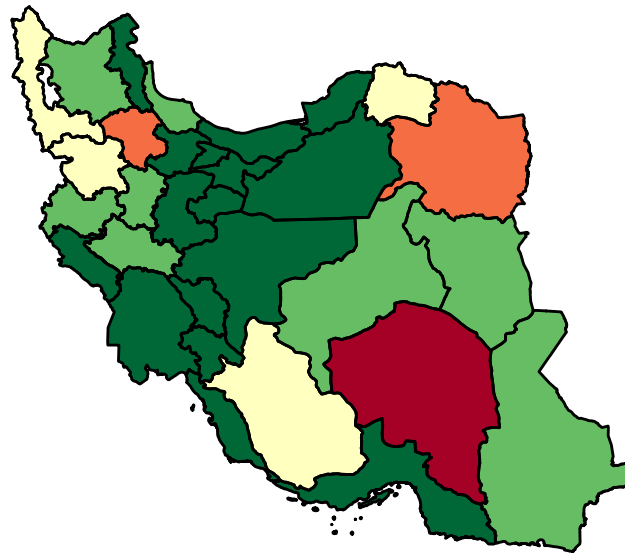

2000

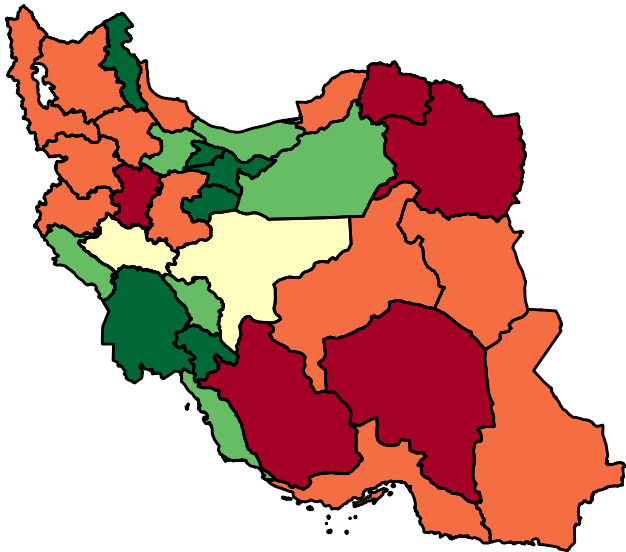

2005

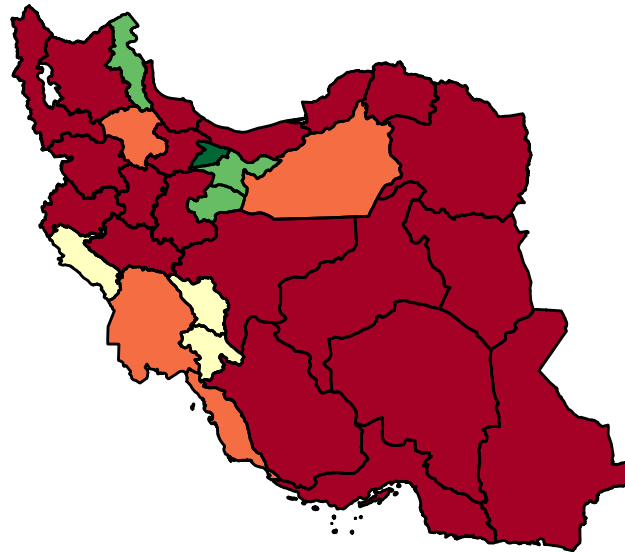

Age standardized  
mortality rate  
(per 100,000)

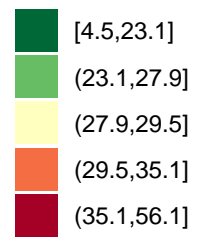

2010

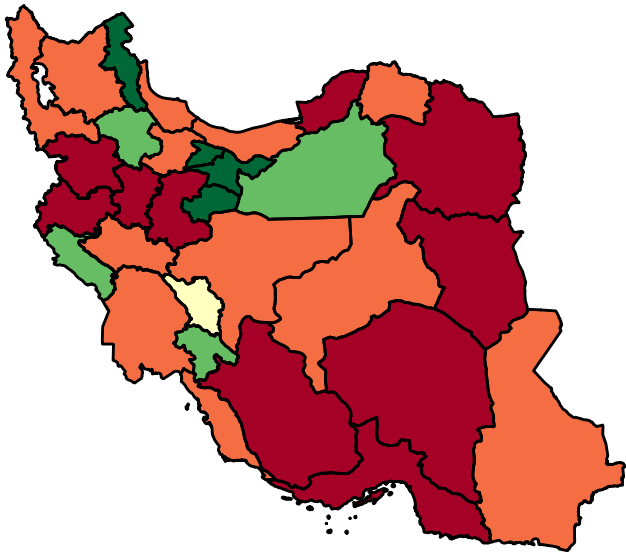

2015

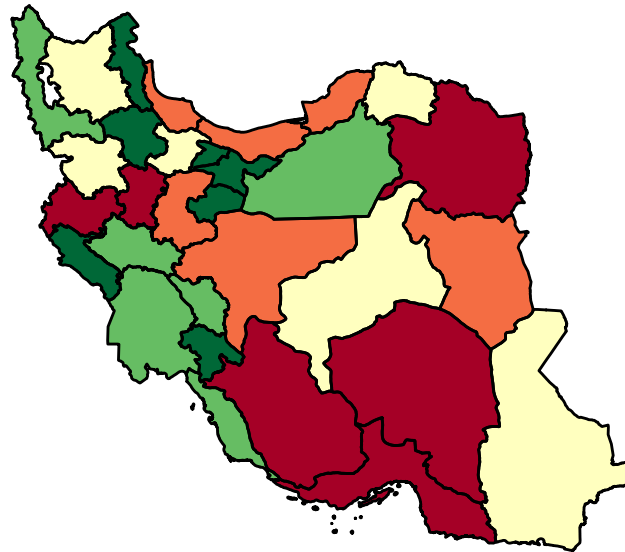

Supplement: Supplementary file 1 — Additional file 1: Supplementary Figure 1. Geographical distribution of age-standardized mortality rate due to road traffic injuries in both sexes in Iran, 1990-2015. Iran’s map is downloaded from https://www.openstreetmap.org/ and further used to draw the figure. [file 12889_2021_11721_MOESM1_ESM.pdf]

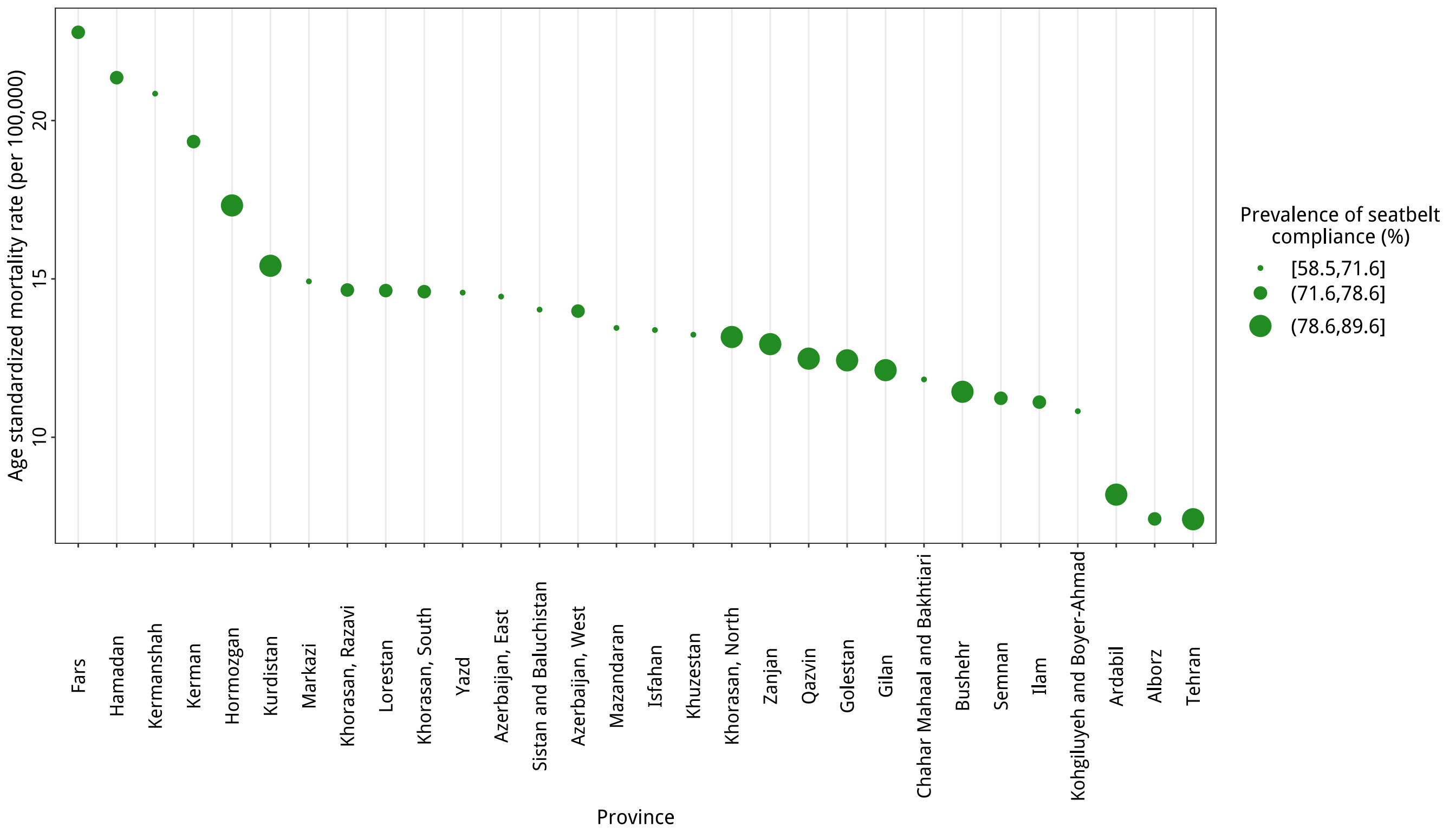

Supplement: Supplementary file 2 — Additional file 2: Supplementary Figure 2. The correlation of age-standardized prevalence of seatbelt usage and RTIs-attributable ASMR caused by motorized vehicle with three-or-more in Iran in both sexes at provincial level. [file 12889_2021_11721_MOESM2_ESM.pdf]

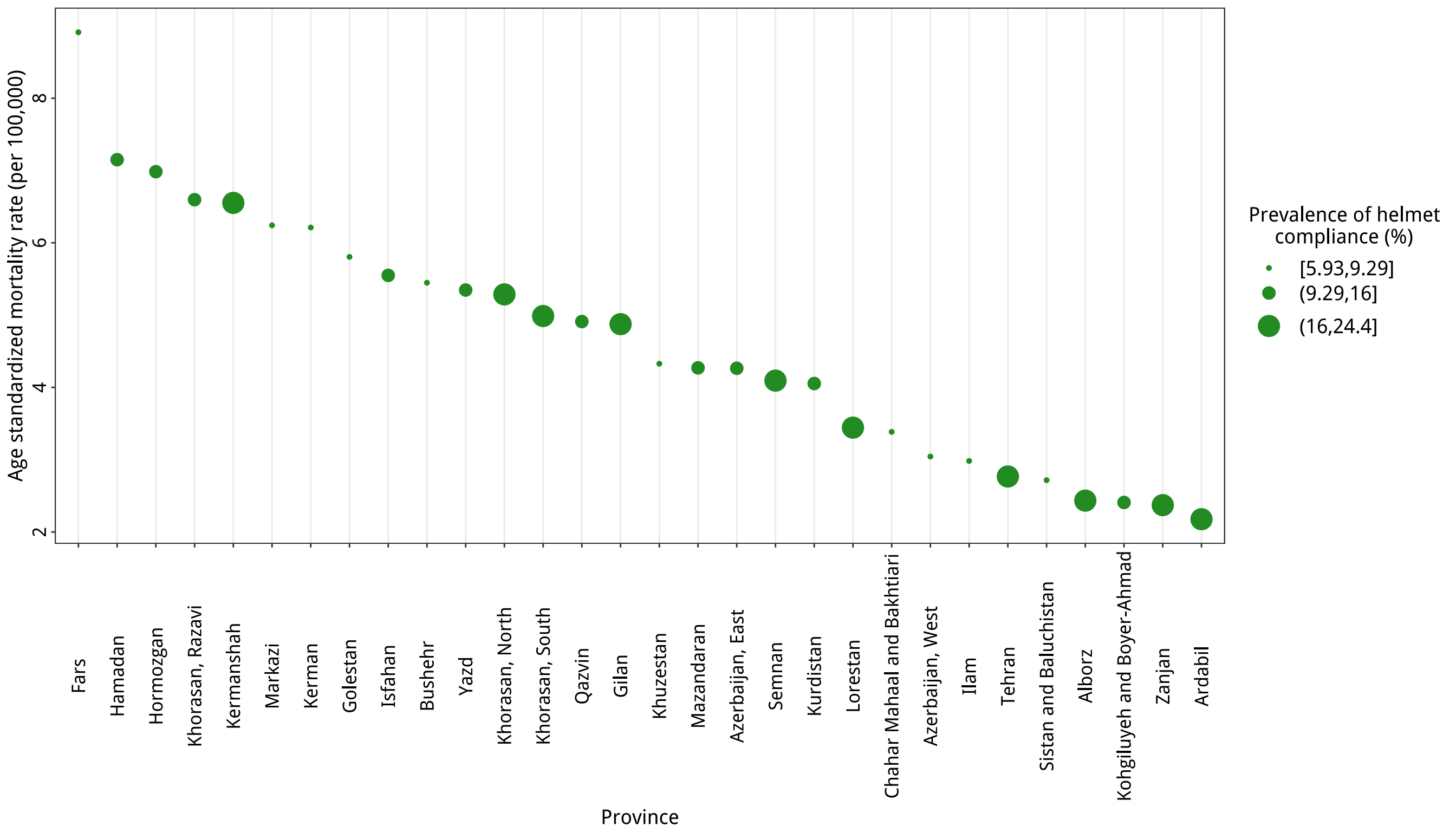

Supplement: Supplementary file 3 — Additional file 3: Supplementary Figure 3. The correlation of age-standardized prevalence of helmet usage and RTIs-attributable ASMR caused by pedal cycle vehicle and motorized vehicle with two wheels in Iran in both sexes at provincial level. [file 12889_2021_11721_MOESM3_ESM.pdf]

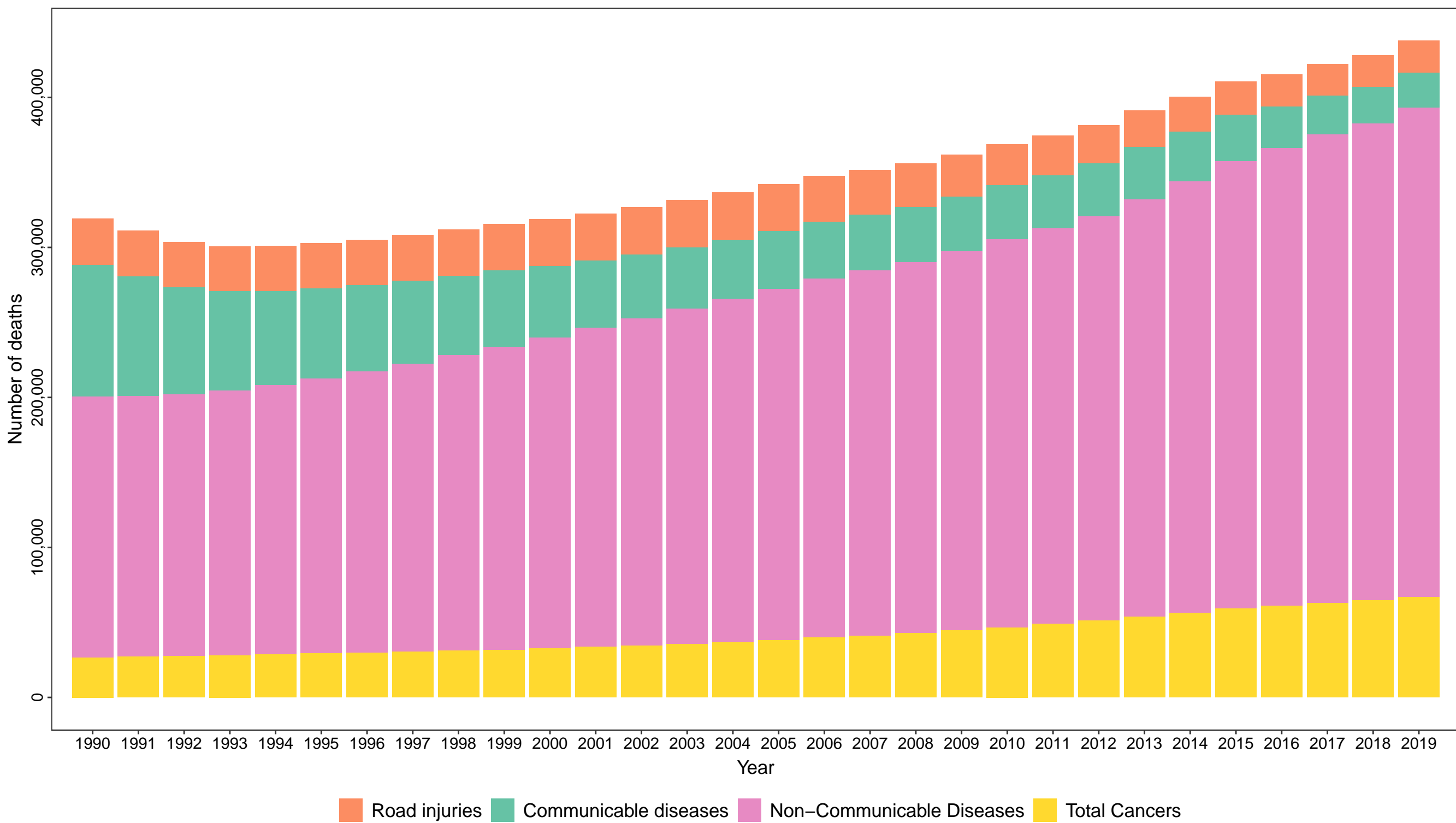

Supplement: Supplementary file 4 — Additional file 4: Supplementary Figure. Adapted from GBD: Comparing number of deaths attributed to non-communicable diseases, communicable diseases, total cancers, and road injuries in Iran from 1990 to 2019 (Data source: http://www.healthdata.org/data-visualization/gbd-compare). [file 12889_2021_11721_MOESM4_ESM.pdf]
