# Supplementary material for: The level and trend of road traffic injuries attributable mortality rate in Iran, 1990–2015: a story of successful regulations and a roadmap to design future policies
Source: BMC Public Health. 2021 Sep 22;21:1722. doi: 10.1186/s12889-021-11721-9 (PMC8459502; doi:10.1186/s12889-021-11721-9)
Supplement: Supplementary file 5 — Additional file 5: Supplementary Table 1. The number of death and age-standardized mortality rates due to RTI by sex, in provinces of Iran, 1990-2015. [file 12889_2021_11721_MOESM5_ESM.docx]

| Supplementary Table 1: The number of death and age-standardized mortality rates due to RTI by sex, in provinces of Iran, 1990-2015 | | | | | | | | | | | | | |
| --- | --- | --- | --- | --- | --- | --- | --- | --- | --- | --- | --- | --- | --- |
| Province | | 1990 | | 1995 | | 2000 | | 2005 | | 2010 | | 2015 | |
|  |  | ASMR per 100,000 (95% UI) | Number of deaths | ASMR per 100,000 (95% UI) | Number of deaths | ASMR per 100,000 (95% UI) | Number of deaths | ASMR per 100,000 (95% UI) | Number of deaths | ASMR per 100,000 (95% UI) | Number of deaths | ASMR per 100,000 (95% UI) | Number of deaths |
| Markazi | Female | 6.48 (5.01-8.4) | 29 | 11.07 (8.73-14.01) | 55 | 14.17 (11.44-17.56) | 77 | 15.76 (12.8-19.37) | 95 | 14.01 (11.35-17.26) | 96 | 10.82 (8.66-13.48) | 86 |
|  | Male | 13.73 (10.66-17.78) | 68 | 28.34 (22.39-35.95) | 145 | 47.1 (38.07-58.24) | 260 | 70.06 (56.88-86.21) | 448 | 65.68 (53.08-81.07) | 466 | 58.68 (47.02-73.06) | 468 |
|  | Both | 10.24 (7.94-13.26) | 97 | 19.8 (15.63-25.1) | 200 | 30.71 (24.81-37.99) | 337 | 43.25 (35.11-53.21) | 543 | 40.26 (32.55-49.68) | 562 | 35.14 (28.16-43.75) | 554 |
| Gilan | Female | 9.01 (6.91-11.75) | 78 | 12.91 (10.07-16.52) | 119 | 14.68 (11.71-18.38) | 147 | 14.04 (11.23-17.53) | 161 | 10.65 (8.47-13.38) | 138 | 9.04 (7.13-11.42) | 136 |
|  | Male | 19.4 (15.07-25.08) | 183 | 34.82 (27.53-44.1) | 335 | 48.17 (39.04-59.55) | 498 | 65.23 (52.54-81.03) | 767 | 57.97 (46.38-72.2) | 741 | 54.3 (43.21-67.97) | 770 |
|  | Both | 14.32 (11.07-18.57) | 261 | 23.82 (18.76-30.26) | 453 | 31.32 (25.28-38.84) | 645 | 39.56 (31.81-49.19) | 927 | 34.22 (27.34-42.68) | 879 | 31.52 (25.05-39.51) | 906 |
| Mazandaran | Female | 8.26 (6.27-10.88) | 78 | 12.21 (9.42-15.85) | 122 | 13.11 (10.25-16.78) | 145 | 13.98 (10.88-18.01) | 177 | 10.72 (8.28-13.84) | 160 | 8.13 (6.22-10.61) | 145 |
|  | Male | 17.01 (13.18-22.05) | 172 | 31.02 (24.36-39.53) | 335 | 41.77 (33.31-52.33) | 498 | 61.06 (47.91-77.64) | 848 | 55.77 (43.54-71.33) | 863 | 51.72 (39.85-67.02) | 904 |
|  | Both | 12.71 (9.79-16.57) | 250 | 21.6 (16.88-27.67) | 457 | 27.4 (21.74-34.5) | 644 | 37.76 (29.58-48.14) | 1025 | 33.45 (26.07-42.85) | 1023 | 29.99 (23.09-38.89) | 1050 |
| East Azarbaijan | Female | 8.27 (6.38-10.72) | 108 | 13.66 (10.74-17.39) | 180 | 15.99 (12.77-20.02) | 226 | 14.58 (11.76-18.06) | 232 | 12.65 (10.19-15.67) | 224 | 9.52 (7.63-11.83) | 190 |
|  | Male | 15.49 (11.92-20.19) | 219 | 33.37 (26.2-42.62) | 472 | 48.87 (39.15-61.08) | 729 | 59.55 (47.96-73.9) | 1008 | 54.97 (44.38-68.07) | 1005 | 48.39 (38.97-59.96) | 956 |
|  | Both | 12.06 (9.29-15.69) | 327 | 23.89 (18.76-30.47) | 652 | 32.87 (26.31-41.1) | 955 | 37.6 (30.29-46.66) | 1240 | 34.11 (27.53-42.25) | 1229 | 29.02 (23.36-35.97) | 1146 |
| West Azerbaijan | Female | 10.26 (7.86-13.4) | 82 | 15.38 (12.11-19.59) | 142 | 14.44 (11.61-17.96) | 152 | 13.14 (10.7-16.13) | 158 | 10.24 (8.34-12.57) | 143 | 7.36 (5.95-9.09) | 119 |
|  | Male | 22.21 (16.87-29.37) | 199 | 40.52 (31.74-51.92) | 405 | 50.68 (40.77-63.05) | 561 | 58.1 (47.34-71.36) | 734 | 53.06 (43.3-64.8) | 748 | 44.95 (36.52-55.15) | 716 |
|  | Both | 16.48 (12.55-21.71) | 281 | 28.34 (22.24-36.26) | 547 | 32.99 (26.54-41.04) | 712 | 36.06 (29.38-44.29) | 892 | 31.87 (25.99-38.95) | 891 | 26.1 (21.19-32.05) | 835 |
| Kermanshah | Female | 8.35 (6.52-10.68) | 57 | 14.89 (11.81-18.76) | 102 | 16.65 (13.4-20.69) | 116 | 19.44 (15.81-23.81) | 152 | 17.62 (14.36-21.61) | 157 | 14.38 (11.64-17.73) | 148 |
|  | Male | 17.89 (14.13-22.77) | 143 | 35.33 (28.31-44.26) | 270 | 49.81 (40.43-61.4) | 379 | 76.1 (62.11-93.36) | 647 | 77.71 (63.44-95.03) | 722 | 70.72 (57.46-86.89) | 732 |
|  | Both | 13.39 (10.54-17.07) | 200 | 25.55 (20.41-32.07) | 372 | 33.77 (27.35-41.72) | 496 | 48.43 (39.49-59.41) | 799 | 47.95 (39.12-58.68) | 878 | 42.5 (34.5-52.25) | 880 |
| Khuzestan | Female | 4.33 (3.35-5.58) | 47 | 8.14 (6.39-10.37) | 106 | 8.98 (7.14-11.32) | 131 | 10.88 (8.69-13.63) | 177 | 10.7 (8.53-13.38) | 205 | 9.11 (7.22-11.44) | 205 |
|  | Male | 9.91 (7.75-12.68) | 113 | 23.19 (18.31-29.39) | 317 | 34.7 (27.91-43.16) | 548 | 49.95 (39.83-62.58) | 933 | 50.04 (40.01-62.49) | 1023 | 46.77 (37.27-58.58) | 1060 |
|  | Both | 7.18 (5.61-9.22) | 160 | 15.84 (12.49-20.09) | 422 | 22.13 (17.76-27.6) | 678 | 30.92 (24.66-38.73) | 1110 | 30.63 (24.48-38.26) | 1228 | 27.87 (22.19-34.93) | 1265 |
| Fars | Female | 8.59 (6.57-11.26) | 127 | 14.78 (11.52-18.93) | 230 | 17.4 (13.82-21.89) | 286 | 20.62 (16.5-25.67) | 376 | 19.93 (15.91-24.88) | 415 | 17.05 (13.49-21.49) | 420 |
|  | Male | 20.08 (15.79-25.59) | 297 | 42.05 (33.47-52.79) | 661 | 62.49 (50.5-77.31) | 1069 | 91.31 (73.46-113.3) | 1841 | 91.49 (73.46-113.89) | 2023 | 81.84 (65.23-102.4) | 2025 |
|  | Both | 14.5 (11.31-18.62) | 424 | 28.62 (22.66-36.12) | 891 | 40.3 (32.45-50.05) | 1355 | 56.7 (45.57-70.4) | 2218 | 56.12 (45-69.91) | 2439 | 49.37 (39.29-61.85) | 2445 |
| Kerman | Female | 12.37 (9.75-15.69) | 86 | 20.85 (16.74-25.92) | 157 | 22.86 (18.67-27.96) | 199 | 20.33 (16.64-24.8) | 211 | 16.03 (13.01-19.68) | 197 | 11.72 (9.41-14.56) | 180 |
|  | Male | 30.57 (24.28-38.49) | 215 | 56.65 (45.77-70.26) | 446 | 85.68 (70.63-103.94) | 788 | 94.73 (77.72-115.35) | 1097 | 80.65 (65.75-98.81) | 1072 | 66.96 (54.03-82.66) | 1037 |
|  | Both | 21.63 (17.14-27.3) | 301 | 38.91 (31.38-48.29) | 603 | 54.87 (45.14-66.68) | 986 | 58.44 (47.92-71.19) | 1308 | 48.73 (39.7-59.73) | 1269 | 39.17 (31.58-48.4) | 1217 |
| Khorasan Razavi | Female | 11.95 (9.08-15.75) | 213 | 19.8 (15.26-25.76) | 378 | 21.15 (16.62-26.95) | 444 | 24.73 (19.42-31.43) | 586 | 15.46 (12.1-19.71) | 420 | 10.35 (8.04-13.29) | 328 |
|  | Male | 19.63 (14.9-25.95) | 384 | 39.54 (30.57-51.27) | 776 | 62.29 (49.25-79.05) | 1328 | 92.52 (72.74-117.59) | 2299 | 74.5 (58.44-95.01) | 2058 | 60.41 (47.07-77.21) | 1864 |
|  | Both | 16.01 (12.16-21.15) | 597 | 29.92 (23.11-38.84) | 1154 | 41.95 (33.11-53.29) | 1771 | 59.04 (46.4-75.04) | 2885 | 45.08 (35.34-57.5) | 2478 | 35.15 (27.37-44.95) | 2192 |
| Isfahan | Female | 7.51 (5.57-10.15) | 118 | 12.41 (9.36-16.44) | 208 | 12.82 (9.82-16.78) | 223 | 13.77 (10.6-17.88) | 270 | 12.13 (9.33-15.74) | 276 | 9.76 (7.46-12.75) | 264 |
|  | Male | 14.03 (10.58-18.63) | 220 | 31.25 (24.01-40.79) | 525 | 42.74 (33.32-54.82) | 797 | 59.61 (46.27-76.76) | 1305 | 56.2 (43.56-72.25) | 1365 | 52.55 (40.52-67.83) | 1430 |
|  | Both | 10.94 (8.21-14.61) | 337 | 22.21 (16.98-29.1) | 732 | 28.3 (21.97-36.45) | 1021 | 37.45 (29.03-48.31) | 1575 | 34.65 (26.81-44.62) | 1641 | 31.34 (24.13-40.53) | 1694 |
| Sistan and Baluchistan | Female | 12.3 (8.9-17.05) | 78 | 18.97 (13.97-25.76) | 134 | 21.76 (16.43-28.79) | 164 | 19.03 (14.5-24.89) | 165 | 15.28 (11.65-19.95) | 148 | 11.42 (8.73-14.91) | 130 |
|  | Male | 21.72 (16.13-29.48) | 135 | 35.62 (26.85-47.59) | 245 | 46.88 (36.37-60.52) | 355 | 51.98 (40.19-67.28) | 453 | 47.39 (36.62-61.26) | 456 | 45.73 (35.38-58.84) | 487 |
|  | Both | 17.03 (12.54-23.28) | 213 | 27.58 (20.65-37.04) | 379 | 34.86 (26.83-45.35) | 519 | 36.29 (27.94-47.12) | 618 | 31.55 (24.3-40.9) | 604 | 28.07 (21.65-36.24) | 618 |
| Kurdistan | Female | 10.24 (7.62-13.72) | 51 | 16.1 (12.44-20.84) | 85 | 15.68 (12.53-19.61) | 86 | 15.78 (12.91-19.27) | 95 | 12.01 (9.84-14.61) | 82 | 8.73 (7.11-10.7) | 68 |
|  | Male | 22.9 (16.63-31.61) | 117 | 41.51 (31.51-54.76) | 229 | 52.4 (41.58-66.12) | 304 | 65.98 (53.79-80.85) | 423 | 60.58 (49.84-73.56) | 425 | 50.12 (41.11-61.06) | 394 |
|  | Both | 16.93 (12.38-23.18) | 168 | 29.32 (22.36-38.49) | 314 | 34.57 (27.47-43.53) | 390 | 41.47 (33.83-50.8) | 518 | 36.59 (30.08-44.45) | 506 | 29.51 (24.18-35.98) | 462 |
| Hamadan | Female | 7.08 (5.57-9) | 48 | 12.09 (9.69-15.07) | 82 | 14.05 (11.45-17.22) | 96 | 15.98 (13.14-19.4) | 118 | 14.69 (12.06-17.87) | 122 | 11.88 (9.66-14.56) | 115 |
|  | Male | 18.22 (14.36-23.18) | 120 | 37.07 (29.68-46.36) | 250 | 57.8 (47.19-70.88) | 404 | 81.21 (66.84-98.56) | 630 | 82.94 (68.04-100.65) | 705 | 76.8 (62.61-93.88) | 737 |
|  | Both | 12.92 (10.18-16.44) | 169 | 24.83 (19.88-31.03) | 331 | 36.1 (29.46-44.26) | 501 | 48.82 (40.17-59.26) | 747 | 49.03 (40.22-59.53) | 827 | 44.67 (36.41-54.62) | 851 |
| Chahar Mahaal and Bakhtiari | Female | 7.1 (5.51-9.15) | 21 | 11.17 (8.81-14.16) | 31 | 10.59 (8.47-13.23) | 31 | 11.01 (8.85-13.64) | 36 | 10.67 (8.61-13.23) | 40 | 8.58 (6.86-10.71) | 39 |
|  | Male | 14.15 (10.88-18.48) | 44 | 25.52 (19.96-32.69) | 76 | 37.4 (29.96-46.67) | 119 | 46.33 (37.47-57.31) | 172 | 45.17 (36.38-55.88) | 186 | 40.6 (32.52-50.55) | 188 |
|  | Both | 10.83 (8.35-14.08) | 65 | 18.52 (14.52-23.65) | 107 | 24.19 (19.37-30.2) | 150 | 28.89 (23.34-35.76) | 208 | 28.09 (22.64-34.77) | 226 | 24.74 (19.81-30.8) | 226 |
| Lorestan | Female | 9.02 (6.88-11.82) | 49 | 13.94 (10.94-17.77) | 81 | 14.23 (11.44-17.68) | 87 | 15.12 (12.39-18.4) | 103 | 12.07 (9.92-14.64) | 92 | 8.19 (6.67-10.01) | 73 |
|  | Male | 19.29 (14.66-25.44) | 129 | 34.54 (27.04-44.32) | 225 | 42.71 (34.4-53.05) | 282 | 62.09 (50.78-75.77) | 465 | 54.34 (44.75-65.93) | 441 | 43.87 (35.8-53.59) | 398 |
|  | Both | 14.28 (10.87-18.79) | 178 | 24.44 (19.15-31.29) | 306 | 28.71 (23.11-35.66) | 369 | 39.06 (31.95-47.64) | 568 | 33.33 (27.44-40.45) | 533 | 25.98 (21.2-31.75) | 471 |
| Ilam | Female | 8.33 (6.31-10.99) | 17 | 13.7 (10.54-17.8) | 26 | 14.54 (11.37-18.64) | 28 | 14.43 (11.35-18.32) | 30 | 11.76 (9.24-14.93) | 27 | 9.33 (7.3-11.89) | 25 |
|  | Male | 11.98 (9.07-15.88) | 23 | 23.66 (18.25-30.72) | 46 | 34.2 (26.84-43.67) | 70 | 42.33 (33.51-53.51) | 103 | 40.24 (31.73-50.94) | 105 | 36.56 (28.66-46.53) | 109 |
|  | Both | 10.33 (7.83-13.67) | 40 | 18.94 (14.61-24.59) | 72 | 24.85 (19.49-31.76) | 98 | 28.87 (22.82-36.53) | 132 | 26.34 (20.76-33.38) | 132 | 23.09 (18.1-29.4) | 135 |
| Kohgiluyeh and Boyer-Ahmad | Female | 9.62 (7.01-13.19) | 19 | 14.8 (11.04-19.84) | 29 | 15.02 (11.4-19.74) | 30 | 15.09 (11.64-19.55) | 34 | 12.42 (9.67-15.95) | 32 | 8.85 (6.89-11.34) | 28 |
|  | Male | 12.12 (8.77-16.82) | 23 | 21.85 (16.22-29.49) | 44 | 30.9 (23.64-40.43) | 68 | 41.57 (32.21-53.55) | 110 | 38.91 (30.48-49.75) | 114 | 33.29 (25.95-42.55) | 114 |
|  | Both | 10.93 (7.93-15.1) | 41 | 18.36 (13.66-24.72) | 73 | 23.09 (17.61-30.26) | 98 | 28.69 (22.19-37.03) | 144 | 25.86 (20.22-33.12) | 146 | 21.12 (16.45-27.02) | 142 |
| Bushehr | Female | 5.6 (4.28-7.32) | 14 | 9.98 (7.85-12.72) | 26 | 11.96 (9.57-14.93) | 34 | 12.16 (9.78-15.09) | 39 | 11.1 (8.85-13.89) | 42 | 9.47 (7.43-12.08) | 46 |
|  | Male | 11.29 (8.78-14.58) | 35 | 23.95 (18.91-30.39) | 70 | 39.59 (31.93-49.09) | 129 | 52.95 (42.72-65.58) | 217 | 51.3 (41.01-64.09) | 250 | 40.61 (31.68-52.02) | 290 |
|  | Both | 8.5 (6.57-11.01) | 48 | 16.98 (13.39-21.57) | 96 | 26.13 (21.04-32.45) | 163 | 33.78 (27.24-41.85) | 256 | 32.86 (26.27-41.05) | 292 | 27.44 (21.46-35.08) | 336 |
| Zanjan | Female | 15.02 (10.95-20.57) | 58 | 20.4 (15.79-26.36) | 76 | 16.51 (13.24-20.54) | 61 | 13.03 (10.43-16.23) | 53 | 8.57 (6.61-11.07) | 39 | 5.32 (3.96-7.11) | 29 |
|  | Male | 29.22 (20.79-41.13) | 101 | 46.6 (35.65-61.1) | 168 | 45.23 (36.01-56.87) | 173 | 55.03 (44.22-68.55) | 238 | 46.92 (36.75-59.77) | 225 | 36.37 (27.78-47.51) | 198 |
|  | Both | 22.24 (15.97-31.04) | 159 | 33.63 (25.82-43.89) | 244 | 30.9 (24.66-38.73) | 233 | 34.04 (27.33-42.4) | 290 | 27.74 (21.68-35.42) | 264 | 20.97 (15.97-27.47) | 226 |
| Semnan | Female | 8.42 (6.25-11.31) | 18 | 12.54 (9.49-16.53) | 27 | 12.72 (9.81-16.53) | 29 | 13.15 (10.25-16.87) | 33 | 11.15 (8.67-14.27) | 33 | 9.38 (7.28-12.05) | 33 |
|  | Male | 11.77 (8.77-15.88) | 23 | 23.4 (17.75-30.97) | 50 | 34.04 (26.3-44.11) | 82 | 46.74 (36.34-60) | 130 | 43.47 (33.84-55.77) | 134 | 40.72 (31.5-52.43) | 142 |
|  | Both | 10.15 (7.55-13.67) | 42 | 18.16 (13.77-24) | 77 | 23.74 (18.33-30.78) | 111 | 30.49 (23.72-39.15) | 164 | 27.61 (21.49-35.4) | 167 | 25.01 (19.36-32.19) | 175 |
| Yazd | Female | 7.6 (5.84-9.92) | 21 | 11.98 (9.31-15.37) | 38 | 13.03 (10.26-16.53) | 47 | 13.31 (10.55-16.76) | 55 | 11.81 (9.36-14.88) | 57 | 9.48 (7.48-11.97) | 57 |
|  | Male | 20.41 (15.82-26.38) | 64 | 39.13 (30.76-49.86) | 132 | 50.5 (40.25-63.47) | 197 | 60.06 (47.87-75.36) | 283 | 53.59 (42.67-67.17) | 278 | 48.92 (38.67-61.6) | 287 |
|  | Both | 14.37 (11.12-18.61) | 86 | 26.08 (20.45-33.29) | 170 | 32.54 (25.88-40.97) | 244 | 37.87 (30.16-47.55) | 338 | 33.54 (26.68-42.07) | 334 | 29.47 (23.29-37.11) | 344 |
| Hormozgan | Female | 4.66 (3.54-6.15) | 17 | 8.72 (6.77-11.23) | 36 | 12.11 (9.57-15.3) | 57 | 13.97 (11.07-17.64) | 73 | 14.46 (11.33-18.44) | 88 | 13.06 (10.02-16.98) | 99 |
|  | Male | 10.21 (7.99-13.08) | 40 | 24.25 (19.23-30.59) | 106 | 46.39 (37.56-57.32) | 231 | 73.8 (59.07-92.11) | 448 | 60.86 (47.92-77.16) | 412 | 65.83 (50.63-85.29) | 525 |
|  | Both | 7.51 (5.83-9.7) | 57 | 16.71 (13.19-21.19) | 142 | 29.94 (24.12-37.16) | 288 | 45.21 (36.12-56.54) | 521 | 38.33 (30.14-48.66) | 501 | 39.53 (30.39-51.26) | 624 |
| Tehran | Female | 5.98 (3.89-9.18) | 225 | 8.29 (5.54-12.42) | 310 | 7.92 (5.47-11.52) | 322 | 9.22 (6.42-13.22) | 439 | 7.26 (5.1-10.34) | 406 | 5.46 (3.82-7.79) | 357 |
|  | Male | 13.5 (9.17-20) | 517 | 23.71 (16.42-34.5) | 937 | 30.02 (21.28-42.41) | 1344 | 41.88 (29.57-59.4) | 2247 | 35.54 (25.27-49.99) | 2132 | 28.88 (20.44-40.73) | 1946 |
|  | Both | 9.87 (6.63-14.78) | 742 | 16.27 (11.17-23.84) | 1246 | 19.35 (13.65-27.5) | 1666 | 26.12 (18.39-37.12) | 2686 | 21.61 (15.33-30.46) | 2538 | 17.09 (12.08-24.16) | 2303 |
| Ardabil | Female | 8.29 (6.34-10.85) | 38 | 13.18 (10.35-16.76) | 60 | 13.15 (10.55-16.36) | 62 | 11.78 (9.58-14.45) | 59 | 9.61 (7.8-11.8) | 53 | 6.94 (5.6-8.6) | 43 |
|  | Male | 12.71 (9.72-16.68) | 62 | 24.87 (19.59-31.67) | 123 | 30.48 (24.59-37.83) | 153 | 40.18 (32.67-49.35) | 218 | 34.02 (27.68-41.75) | 199 | 29.74 (24-36.8) | 192 |
|  | Both | 10.57 (8.09-13.85) | 100 | 19.15 (15.08-24.36) | 183 | 21.88 (17.63-27.17) | 215 | 26.1 (21.23-32.05) | 277 | 21.95 (17.85-26.95) | 252 | 18.51 (14.94-22.91) | 235 |
| Qom | Female | 5.82 (4.14-8.2) | 13 | 8.92 (6.46-12.34) | 24 | 10.62 (7.82-14.4) | 34 | 11.45 (8.51-15.38) | 44 | 6.72 (5.01-9) | 32 | 5.77 (4.31-7.71) | 33 |
|  | Male | 15.78 (11.32-22.08) | 41 | 26.97 (19.74-37.02) | 85 | 33.37 (24.9-44.85) | 127 | 36.87 (27.66-49.21) | 169 | 22.25 (16.7-29.59) | 116 | 19.35 (14.54-25.79) | 117 |
|  | Both | 11.02 (7.89-15.44) | 54 | 18.27 (13.34-25.13) | 110 | 22.38 (16.65-30.15) | 161 | 24.63 (18.44-32.94) | 213 | 14.74 (11.05-19.64) | 148 | 12.73 (9.55-16.98) | 150 |
| Qazvin | Female | 9.06 (7.12-11.57) | 41 | 13.76 (10.97-17.25) | 54 | 14.96 (12.1-18.45) | 59 | 15.78 (12.89-19.29) | 72 | 12.59 (10.24-15.42) | 67 | 9.75 (7.87-12.05) | 62 |
|  | Male | 15.62 (12.3-19.91) | 65 | 30.35 (24.24-38.07) | 120 | 38.67 (31.35-47.74) | 167 | 64.83 (52.93-79.52) | 330 | 56.41 (45.96-69.11) | 323 | 46.84 (37.8-57.89) | 304 |
|  | Both | 12.45 (9.8-15.88) | 106 | 22.26 (17.77-27.91) | 174 | 27.11 (21.96-33.45) | 226 | 41.03 (33.5-50.3) | 402 | 34.94 (28.45-42.8) | 390 | 28.42 (22.94-35.12) | 366 |
| Golestan | Female | 7.33 (5.66-9.47) | 37 | 12.63 (9.95-16) | 64 | 14.42 (11.55-18.01) | 77 | 14.31 (11.5-17.76) | 87 | 12.47 (9.98-15.54) | 90 | 10.66 (8.44-13.44) | 94 |
|  | Male | 13.43 (10.25-17.66) | 65 | 29.23 (22.72-37.7) | 152 | 47.36 (37.55-59.75) | 276 | 71.54 (57.39-89.2) | 491 | 64.94 (51.71-81.44) | 512 | 53.95 (42.37-68.39) | 483 |
|  | Both | 10.49 (8.04-13.71) | 102 | 20.98 (16.37-26.92) | 216 | 30.88 (24.54-38.87) | 352 | 42.93 (34.45-53.49) | 578 | 38.72 (30.86-48.51) | 603 | 32.3 (25.4-40.91) | 577 |
| North Khorasan | Female | 10.04 (7.42-13.61) | 25 | 15.91 (12.07-20.98) | 42 | 16.21 (12.57-20.93) | 46 | 15.89 (12.49-20.17) | 51 | 13.12 (10.31-16.64) | 49 | 9.73 (7.61-12.39) | 44 |
|  | Male | 22.7 (16.42-31.51) | 59 | 39.82 (29.73-53.57) | 110 | 54.87 (42.32-71.25) | 164 | 66.52 (52.28-84.53) | 226 | 56.12 (44.23-71.03) | 215 | 49 (38.46-62.17) | 218 |
|  | Both | 16.69 (12.15-23.02) | 84 | 28.04 (21.02-37.52) | 152 | 35.57 (27.47-46.15) | 210 | 41.08 (32.28-52.21) | 277 | 34.43 (27.12-43.6) | 264 | 29.31 (22.99-37.21) | 262 |
| South Khorasan | Female | 9.23 (6.84-12.46) | 24 | 14.77 (11.2-19.5) | 36 | 16.73 (12.9-21.66) | 43 | 16.16 (12.57-20.72) | 47 | 14.43 (11.23-18.51) | 45 | 11.4 (8.84-14.64) | 41 |
|  | Male | 20.36 (14.94-27.86) | 60 | 35.84 (26.93-47.81) | 91 | 51.68 (39.93-67.08) | 136 | 61.82 (48.3-79.15) | 184 | 58.19 (45.5-74.43) | 181 | 50.06 (38.9-64.11) | 171 |
|  | Both | 15.06 (11.09-20.53) | 84 | 25.45 (19.17-33.85) | 127 | 34.36 (26.53-44.58) | 179 | 39.29 (30.66-50.33) | 230 | 36.39 (28.42-46.58) | 227 | 30.73 (23.88-39.38) | 211 |
| Alborz | Female | 2.67 (1.95-3.67) | 10 | 4.66 (3.47-6.26) | 22 | 6.49 (4.92-8.58) | 40 | 7.46 (5.7-9.76) | 61 | 7.65 (5.86-9.96) | 79 | 6.67 (5.08-8.76) | 86 |
|  | Male | 6.21 (4.62-8.38) | 29 | 13.04 (9.85-17.3) | 71 | 19.88 (15.21-26) | 135 | 27.36 (21.07-35.52) | 244 | 28.35 (21.89-36.77) | 313 | 26.77 (20.41-35.01) | 361 |
|  | Both | 4.53 (3.36-6.14) | 39 | 9.06 (6.82-12.06) | 93 | 13.5 (10.31-17.7) | 175 | 17.85 (13.72-23.22) | 305 | 18.27 (14.08-23.72) | 391 | 16.76 (12.77-21.94) | 447 |
| National | Female | 8.14 (6.1-10.9) | 1849 | 12.93 (9.9-16.91) | 3081 | 13.95 (10.91-17.88) | 3577 | 14.72 (11.56-18.76) | 4289 | 12.01 (9.42-15.31) | 4052 | 9.31 (7.25-11.94) | 3722 |
|  | Male | 16.78 (12.67-22.36) | 3964 | 32.29 (24.91-42.06) | 8018 | 45.34 (35.78-57.62) | 12369 | 61.61 (48.56-78.28) | 19709 | 55.64 (43.86-70.61) | 19809 | 48.77 (38.19-62.19) | 19622 |
|  | Both | 12.64 (9.52-16.86) | 5812 | 22.87 (17.61-29.82) | 11099 | 30.01 (23.63-38.21) | 15946 | 38.74 (30.51-49.26) | 23998 | 34.13 (26.88-43.35) | 23861 | 29.1 (22.76-37.14) | 23344 |
